# Is There a Place for Responsible Artificial Intelligence in Pandemics? A Tale of Two Countries

**DOI:** 10.1007/s10796-021-10140-w

**Published:** 2021-05-06

**Authors:** Ramzi El-Haddadeh, Adam Fadlalla, Nitham M. Hindi

**Affiliations:** College of Business and Economics, Qatar University, P.O. Box 2713, Doha, Qatar

**Keywords:** Responsible Artificial Intelligence, Ethics, Sentiment analysis, COVID-19 pandemic

## Abstract

This research examines the considerations of responsible Artificial Intelligence in the deployment of AI-based COVID-19 digital proximity tracking and tracing applications in two countries; the State of Qatar and the United Kingdom. Based on the alignment level analysis with the Good AI Society’s framework and sentiment analysis of official tweets, the diagnostic analysis resulted in contrastive findings for the two applications. While the application EHTERAZ (Arabic for precaution) in Qatar has fallen short in adhering to the responsible AI requirements, it has contributed significantly to controlling the pandemic. On the other hand, the UK’s NHS COVID-19 application has exhibited limited success in fighting the virus despite relatively abiding by these requirements. This underlines the need for obtaining a practical and contextual view for a comprehensive discourse on responsible AI in healthcare. Thereby offering necessary guidance for striking a balance between responsible AI requirements and managing pressures towards fighting the pandemic.

## Introduction

Artificial Intelligence (AI) technologies have become a driving force for transforming businesses and societies and have been profoundly reshaping our lives and ecosystem (Daugherty et al., 2019,  Choi et al., 2017). Understanding AI’s impact on societies became vital to demonstrate its capabilities and value. Such innovative technology has demonstrated its potential to positively contribute to their growth and improve the overall quality of life (Schönberger, 2019). However, some apprehensive opinions, worries and securities accompanied AI technologies battling perception issues, i.e. man vs. machine (Wilson & Daugherty, 2018). Therefore, examining the regulatory, governance, and ethical perspectives of AI became necessary to assess its maturity and offerings to tackle various societal challenges, including improving well-being, justice, sustainability, and resilience (Floridi et al., 2018; Schönberger, 2019). As such, responsible AI emerged to offer the mechanisms for integrating AI technologies in an ethical, transparent and accountable manner to nurture trust and privacy and mitigate its risks (Ghallab, 2019; Wang et al., 2020).

For digital health technologies, the advancement in healthcare systems and the increase in medical data volume have created AI innovations opportunities. The exploitation of AI technology in healthcare offered new prospects for healthcare providers to develop further in-depth insights towards understanding the causes of different illnesses, medical interventions and associated healthcare activities. Nevertheless, with such fast-paced deployment, there appears to be a limited consideration of AI technology diffusion’s ethical complexities in the health sector (Floridi, 2018; He et al., 2019). Studies have often pointed out the gap between the development and the adoption of AI tools in healthcare, given the fact that these tools have been designed with little appreciation to the ethical perspective (Ienca et al., 2018; Fiske et al., 2019). Besides, Fiske et al. (2019) pointed out that there have been limited considerations for the societal and ethical implications when integrating AI solutions in a clinical setting.

In the context of the COVID-19 pandemic, governments and public health entities have accelerated the deployment of various innovative technological initiatives, including AI-based digital proximity tracking and tracing applications, in their fight to control the spread of the COVID-19 virus. However, there have been some discrepancies in how the application has been adopted across many countries. Such differences were reflected in the design, development, deployment, diffusion, and ethical considerations resulting in some mixed outcomes in their effectiveness. Thus, an argument can be made for the need to closely examine COVID-19 digital proximity tracking and tracing application deployment against their performance in fighting the pandemic.

While it is evident that AI-based technologies offer opportunities across various contexts, the responsible perspective of AI is yet to be appreciated. Hence, this study aims to examine responsible AI’s considerations in deploying COVID-19 digital proximity tracking and tracing health applications in two countries, the State of Qatar and the United Kingdom. These two countries were chosen due to two main reasons, including convenience and studying generality, by selecting countries to maximise diversity along the dimension in question to explore the scope or universality of a phenomenon. From the political perspectives, both countries have distinctive political sittings, in which both the UK and Qatar political system is a constitutional monarchy. While the government and parliament in the UK take political decisions, sharia law is the primary Qatari legislation source. From an economic perspective, both the UK and Qatar has one of the highest GDP per capita and ranking generally among the top 30 wealthiest countries in the world (IMF, 2021). Besides, both countries have a well-established healthcare system that the government publicly funds from a healthcare perspective. To pursue this, this study will utilise secondary data resources to overcome the limitation of direct access to primary empirical data, especially during the pandemic. As such, existing resources, including official documents, official information platforms, and associated standard repositories coupled with official social media engagement sentiment analysis, will help offer the required diagnostic insights and build necessary data triangulation (Kennedy, 2008). This should help better understand how governments, particularly official healthcare service providers, consider the ethical dimension in the design, implementation, deployment and relevant engagement strategies for these AI-based applications during pandemics. Thus, the emerging good AI society framework (Floridi et al., 2018) will be utilised in this study as it is built on several well-established universal bioethics principles and is one of the first leading forums dedicated to discussing AI’s social impact, specifically in the digital health domain (Nakata et al., 2020). Hence, this will offer a responsible AI perspective to verify emerging ethical themes and principles captured through this process.

To address the above research aim, this article first offers a taxonomy on responsible AI in the digital health context in Sect. 2. This will attempt to carefully build a critical understanding of responsible AI’s current status quo in this context. Section 3 presents the elucidates AI-enabled COVID-19 digital proximity tracking and tracing applications to verify related socio-technical issues. Section 4 discusses existing responsible AI frameworks and initiatives with careful attention towards articulating the good AI society framework in the context of AI-based digital proximity tracking and tracing. The methodological approach adopted for this study is presented in Sect. 5, highlighting the contributions of secondary data analysis to research, including the use of sentiment analysis as an emerging phenomenon. The findings of the utilised framework and related sentiment analysis on COVID-19 digital proximity tracking and tracing are then examined in two different contexts, (NHS COVID-19 app) UK and (EHTERAZ) Qatar, in Sect. 6. After that, Sect. 7 discusses the study’s main findings and offer the appropriate synthesis to the extant literature. Finally, Sect. 8 offers relevant conclusions and implications.

## Responsible AI for Digital Health: Literature Taxonomy

The advancement of AI has created the need for realising its risks and allied impact on businesses and societies in various domains (Schönberger, 2019; Fiske et al., 2019). Insights derived from AI models to support and automate decision-making processes require careful considerations covering moral, ethical and legal perspectives. In this context, responsible AI emerged to offer the guidance needed to appreciate the ethical side’s perspective for this innovative technology. Martinez-Martin et al. (2020) argued that appreciating the ethical perspective in any context, including healthcare, should be acknowledged as a whole rather than at an individual level. Furthermore, Dignum (2019) characterised responsible AI to be *" concerned with the fact that decisions and actions taken by intelligent autonomous systems have consequences that can be seen as being of an ethical nature…Responsible AI provides directions for action and can maybe best be seen as a code of behaviour — for AI systems, but, most importantly, for us”*. This definition verifies the significance of ethics for this innovative technology and its decisions across all levels. Therefore, upholding an ethical and responsible outlook of AI and machine learning technologies becomes imperative for AI applications to prevail.

In the context of digital health technologies, the ability for AI algorithms to learn from existing clinical data is offering limitless opportunities for many healthcare providers. This can include improving patients diagnosis, refining medical decision making, offering a robust personalised medicine, as well as enhancing patients experiences (Amato et al., 2013; Bennett & Hauser, 2013; Dilsizian & Siegel, 2014; Chin-Yee & Upshur, 2019; Harerimana et al., 2018; He et al., 2019). Nonetheless, exploitation of these algorithms has raised many concerns in many areas, including the ethical side. In particular, Morley and Floridi (2020) argued that healthcare providers would need to consider the ethical risks associated with AI deployment, given its impact on delivering healthcare services. Furthermore, Challen et al. (2019) and He et al. (2019) claimed that while establishing rules and regulatory measures can offer some governance and compliance mechanisms, observing end-user ethical rights and appreciating the value remains a challenge.

Furthermore, AI has been used in all the significant healthcare domains broadly either by automating tasks or augmenting decision-making (Jiang et al., 2017). Using AI for augmentation has been a preferred approach to integrating humans and intelligent machines for collective and supporting decision-making capabilities (Davenport & Kirby, 2015). Although AI applications in healthcare have accrued excellent resource efficiency and productivity gains (through automation) and improved quality of work and versatility (through augmentation), they also have raised many ethical concerns, including issues of privacy, security, trust, responsibility, and accountability (Murphy et al., 2021). Acknowledging the necessary need for the power of AI and the irreversible tide of its use, many organised and scholarly efforts have been advancing frameworks and strategies (Floridi et al., 2018, Leslie, 2020, Murphy et al., 2021) to mitigate the ethical concerns posed by the applications of AI in healthcare. Nonetheless, the pandemic has added another complicating factor (urgency) to the discourse on the delicate balance between leveraging AI’s power and mitigating ethical concerns. While some countries opted to assign more weight to the use of AI applications to help control the pandemic, even if some sacrifices are made on the ethical aspects, others assigned more weight to the ethical use even if this comes at a slower pace of controlling the spread of the virus. Such debate is still unresolved and will most likely resurface with future pandemics, although hopefully with less intensity as more maturity might be achieved in embedding ethical design in future AI applications due to advancement in ethical AI scholarship practice. Table 1 presents a taxonomy on the main goals of using AI in healthcare, sample applications, ethical concerns, and mitigating strategies.

**Table 1:** Taxonomy on AI goals in healthcare and associated ethical concerns

AI Goals in Healthcare	Application Domains	Ethical Concerns	Mitigating Strategies	References
Automation	• Robot carers for the elderly • Robotic surgery • Wearable medical devices • Ambient intelligence	• Issues of privacy, security, trust, accountability, responsibility, and bias • Issues of social isolation • Issues of human dignity • Issues of autonomy	• A participatory approach to AI development; engagement of end-users and beneficiaries; shared responsibility for all; and appropriate and responsible AI technology governance through regulatory mechanisms and infrastructure (Murphy et al., 2021). • Adopting open science and sharing data responsibly; caring and acting through responsible research and innovation; adopting ethical principles to create a shared vocabulary for balancing and prioritising conflicting values; generating and cultivating public trust through transparency, accountability, and consent; and fostering equitable innovation and protecting the interests of the vulnerable (Leslie, 2020)	Murphy et al. (2021), Paul et al. (2018), Iyengar et al. (2018), DeCamp and Tilburt (2019), Smith (2020), Challen et al. (2019), Parikh et al. (2019), Sorell and Draper (2014), Van Wynsberghe (2016), Martinez-Martin (2020)
Augmentation	• AI-powered diagnosis systems • AI-powered clinical decision support systems • Radiomics and radiology image processing and recognition	• Issues of privacy, security, trust, accountability, responsibility, and bias • Issues of explainability • Disruptions to provider-patient interactions • Issues of gene editing and human genetic choices		Murphy et al. (2021), Paul et al. (2018), Iyengar et al. (2018), DeCamp and Tilburt 2019; Smith (2020), Challen et al. (2019), Parikh et al. (2019), Morley et al. (2020a, b), Rigby (2019), Minari et al. (2018), Fiore and Goodman (2016), Sabatello (2018), Adhikary et al. (2019), Leslie (2020), Schönberger (2019)

The abovementioned challenges have identified the need to develop holistic guidance towards addressing the ethical and responsible perspectives on AI. As such, clinicians, policy and lawmakers, AI specialists, associated end-users entities, and ethics experts will need to work closely towards developing appropriate and applicable frameworks and guidelines to mitigate these identified ethical risks. By doing so, will help in establishing a baseline for those looking to implement healthcare AI-based solutions.

## Elucidating AI-Enabled Covid-19 Digital Proximity Tracking and Tracing

Recent studies identified various AI solutions to tackle some pandemic outbreaks, including Ebola and COVID-19. In this regard, Colubri et al. (2019) verified the potential and applicability of using machine learning algorithms to set up prognostic models for examining the outbreaks of the Ebola epidemic. Similarly, To˘gaçar et al. (2020) deployed artificial deep learning models for examining COVID-19 in chest x-ray images increasing the efficiency in detecting the disease. Furthermore, Choi et al. (2017) offered insights into the benefits of employing AI and machine learning models to better understand outbreaks from a public health perspective. These studies highlight the potential of utilising various machine learning and AI algorithms in examining public health outbreaks.

Nonetheless, the World Health Organisation (WHO), research, clinical communities, and the public health authorities have endeavoured to explore innovative technologies in the fight to control the spread of the COVID-19 virus. This comprised tracing and contacting those infected to isolate and prevent them from infecting others. In this context, attempts have been made to employ AI-based solutions that offered considerable capabilities to fight this pandemic. Specifically, the expectations were focused on providing the needed support for clinical predictions and policy-focused decisions by improving screening (Wong et al., 2019; Char et al., 2020). In the context of the COVID-19 virus spread, AI-enabled digital proximity contact tracing applications appear to be effective tools that can help break the virus’s transmission chain (O’Neill et al., 2020). In principle, they can identify and manage exposed and infected individuals and help avoid the virus to spread. The underlying technology utilised in these applications is based on Bluetooth, Global Positioning System (GPS), Social graph, contact details, network-based Application Programming Interface (API), mobile tracking data, card transaction data, and system physical address (Lalmuanawma et al., 2020). These technologies are often deployed through centralised, decentralised, or even sometimes, a hybrid of both mechanisms, offering an agile approach towards capturing data in real-time. Here, AI’s role focuses on providing much-needed insights and analysis for tracing infected and vulnerable individuals (Wikipedia, 2020; O’Neill et al., 2020).

While such an AI-based tool has demonstrated its capabilities in fighting infections (Rorres et al., 2018), concerns about the contact tracing applications focused on ethics, privacy and data management and control. As such, various countries imposed specific policies, and sometimes laws, in their attempt to successfully deploy these applications in their fight against the pandemic despite these concerns. In some cases, several contact and tracing apps violate privacy laws and are sometimes deemed unsafe (O’Neill et al., 2020). Furthermore, ethical questions concerning these applications have also started to surface. This included questions that revolved around whether they are mandatory or voluntary and those directed towards access control, handling, storing, and disposal of collected data (Lalmuanawma et al., 2020; O’Neill et al., 2020).

## Responsible AI Frameworks and Initiatives

The realisation of ethical and risk implications for AI has attracted momentous attention to offer the necessary guidance for developing and deploying AI applications (Dilsizian & Siegel, 2014; Chin-Yee & Upshur, 2019; Rorres et al., 2018). A limited number of initiatives, recommendations, research studies, principles and strategies were introduced to offer this essential foundation. In this regard, when examining the literature on frameworks of models targeting the topic of AI ethics, or responsible AI, the results appear to be modest. Most of these initiatives have often been focused on establishing various groups/organisations and setting out principle documents and reports to provide necessary guidance within this domain (Murphy et al., 2021; Floridi, 2018). This includes Partnership on AI (https://www.partnershiponai.org/), OpenAI (https://openai.com), Foundation for Responsible Robotics (https://responsiblerobotics.org/), Ethics and Governance of Artificial Intelligence Initiative (https://aiethicsinitiative.org), Montréal Declaration for Responsible Development of Artificial Intelligence (University of Montreal, 2017), Principles for Accountable Algorithms (Jobin et al., 2019), the Good AI Society Framework (AI4people) (Floridi et al., 2018), and Leslie (2019) who attempted to provide some guidelines focused towards the explainability of AI systems designs and implementation from a practical perspective. While the aforementioned suggests the area of ethics and AI appears to be nascent, Floridi et al. (2018) AI4poeple appears to be the most conventional academic framework given it has been built on established bioethics research, specifically in the health sector.

The good AI society framework (AI4People) established by Floridi et al. (2018) has been built on several well-established universal bioethics principles, which have been widely adopted in the area of applied ethics (Floridi, 2013; Beauchamp & Childress, 2001). The AI4People framework is one of the first leading forums dedicated to discussing AI’s societal impact (Nakata et al., 2020). Furthermore, this framework has been acknowledged to protect societal values and principles (Morley et al., 2020a, b). The AI4People framework offers a synthesised and comprehensive five well-rounded principles: beneficence, non-maleficence, autonomy, justice and explicability. The first principle, beneficence, verifies whether the AI application is or can do only good for society. It mainly focuses on understanding how AI can support and promote human well-being throughout these systems’ design. Besides, appreciating how the development of AI can be instrumental in empowering people in order to preserve their dignity. Concurrently, continuing the prosperity of humankind and the protection of the environment for the future. On the other hand, the second principle, non-maleficence, targets the guarantee that AI does not harm humanity. To do so, maintaining personal security and privacy becomes necessary to control and protect personal data and deter any potential misuse of AI. Nonetheless, managing this appropriately requires continuous efforts in promoting a thoughtful understanding of intentional or unintentional harms. For autonomy, the third principle, the attention is directed towards managing decision-making responsibilities. In this regard, individuals will need to be empowered enough to make decisions rather than expecting machines/artificial agents to take them entirely on their behalf. The fourth principle of justice targets the upholding of prosperity and solidarity. To do so, AI will be expected to inspire fairness and stop discrimination from sharing its benefits while being conscious of limiting any deviations to existing social structures that might create some new harms. However, while these four principles have been built on previous well-established bioethics principles, the fifth principle, explicability, focuses on understanding how humankind can establish and build trust with AI from a decision-making capability perspective to realise its value. Therefore, a clear explanation for AI decisions, i.e. intelligibility, and verifying responsibility and accountability, will be required. For the first, it is motivated towards improving the visibility of the AI decision-making process. As for the second, it is more concerned with the ability to audit these decisions.

## Methodology

Given the limited availability of the information resources related to this study, this research utilised two forms of secondary data sources to provide the applied and diagnostic perspective to the developed framework on responsible AI. The first is based on official documents and sources and public media dissemination reports provided through the two digital proximity tracking and tracing apps under study. The second type targets Twitter’s microblogging platform by conducting the appropriate sentiment analysis. Secondary data availability for scientific research has offered opportunities to provide new perspectives on various research problems across many disciplines (Sarker et al., 2020). Hox and Boeije (2005) stated that secondary data could be identified as the exploitation of existing data that has been collected for different purposes to answer a different research problem. Patzer (1995) and Shmueli (2010) highlighted how taking a careful perspective of recent, relevant and accurate data can achieve a high sense of validity and reliability needed for conducting impactful scientific research.

Sarker et al. (2020) argued how, in information systems, primary data collection could be challenging due to financial and human resources constraints. They also claimed that such challenges could be associated when the phenomena being examined are at the macro/country level. As such, it gives a valuable opportunity to support and enable a macro-level validation and view of phenomena being examined at a large scale, such as the country level. Moreover, maintaining a bottom-up approach, as in secondary data exploration, can offer the opportunity to closely examine theories, frameworks and models (Constantiou & Kallinikos, 2015). Similarly, Yin (2009) argued how secondary data could be acknowledged and used as a standard and reliable source of information. Besides, Zhang (2016) claimed how media outlets often offer valuable insights into current trends and approaches derived by government officials. In this regard, a diverse range of secondary sources, including reports and official and formal web content, can offer an in-depth and comprehensive view of these initiatives’ dynamics and associated applications. Hence, offering opportunities to help contribute to the advancement of knowledge (Lazer et al., 2014).

Furthermore, the advancement of innovative technologies and the exponential growth and availability in data, i.e. big data, have offered opportunities for a plethora of analytical tools, applications and methods (McAfee & Brynjolfsson, 2012; Clarke, 2016). It enabled the capturing of unconventional insights, especially for those generated through various social networking outlets, and Natural Language Processing (NLP) method has been recognised as one of these innovative tools (Sun et al., 2017; Li et al., 2015; Collobert et al., 2011). As such, sentiment analysis has emerged as an innovative opinion-mining tool supporting the measurement of opinions, thoughts and behaviours based on textual data (Ji et al., 2015; Liu, 2012). The lexicon-based approach is regarded as one of the main techniques utilised to offer the required sentiment scoring (Sun et al., 2017; Jockers, 2017). Such scores are classified as positive, negative and neutral sentiments, and they are built on a dictionary of words offering the mechanisms to determine data polarity (Sun et al., 2017; Ravi & Ravi, 2015).

Microblogging social networking platforms have attracted a considerable amount of attention for conducting such an analysis. In particular, Twitter has been identified as one of the most popular social networking applications often exploited by an array of diverse users, including consumers, government entities, policymakers, businesses, and decision-makers (Kwak & Grable, 2021). This microblogging platform offered endless opportunities to examine many entities’ attitudes and behaviours, specifically policymakers, to help build strategies for a better understanding and engagement (Kang et al., 2017).

The abovementioned techniques and associated tools offer the opportunity to examine an applied viewpoint to a theoretically developed framework on responsible AI. As such, this is expected to offer much-needed insights regarding how governments, particularly official healthcare service providers, consider the ethical dimension in the design, implementation, and relevant engagement strategies for these AI-based applications during pandemics. For this study, many of these resources included official documents, official information platforms, and associated standard repositories, were utilised. This includes various government websites in the UK and Qatar and publicly available resources, such as official documents and reports and academic reports. Furthermore, Twitter Application Programming Interface (API) has been utilised to apply sentiment analysis for the captured tweets to unfold the engagement tone for both applications and verify the emerging ethical themes captured through this engagement. Such an approach should provide a general perspective on how responsible AI is conceptualised, applied, and practically validated in these natural settings during the pandemic. Thus, this study’s exploitation of the utilised secondary data resources should build necessary data triangulation (Kennedy, 2008).

## Findings and Analysis

### COVID-19 Digital Proximity Tracking: Implementation, Outcome and Impact

Based on the International health regulations guidelines, the World Health Organisation (WHO) identified that all member states are required to develop public health surveillance systems to capture and monitor critical data related to coronavirus infections (World Health Organization, 2020). Simultaneously, these systems will need to preserve transparency, responsiveness and not cause burdens on communities (World Health Organization, 2020). In principle, the process of digital proximity tracking and tracing is to identify and manage individuals who are tested positive and recently exposed to an infected COVID-19 patient. The process involves 14 days of monitoring and follow-ups to avoid further spread and limit the virus’s exposure. As such, many countries have worked on developing mobile applications embedding AI-based technologies. In the United Kingdom context, the NHS identified AI as an integral building block for the NHS (National Health Service, 2019) in England and Wales. It highlights its vision regarding how AI will support clinicians in implementing best practice, maintaining conforming performances throughout the pathway of care, and guiding patients in managing their health. The NHS COVID-19 app has been developed as part of a major initiative referred to as NHS Test and Trace service in England and the NHS Wales Test, Trace, Protect service in Wales. The app has six main features; trace, alert, check-in, symptoms, test and isolate. In this respect, the app allows users to report symptoms and check if symptoms could be related to coronavirus, order a coronavirus test, obtain test result, check in to participating venues by scanning a QR code, and help the NHS trace individuals that have coronavirus^1^. In addition, the app allows users to countdown for how long they will need to self-isolate. The alert feature helps users better understand whether their area has become a high-risk coronavirus area. As far as the technological underpinning for this app, the NHS initially opted for the centralised approach when it launched the alpha and beta versions earlier in April 2020. However, due to various technical challenges in detection, the decision was to move to a decentralised system based on the AI-enabled Apple/Google exposure notification system (Apple, 2020) and relaunched the app in September 2020. From the ethical perspective for this mobile application, the NHS explicitly emphasised how it took all necessary measures to adhere to various privacy, security and ethical requirements. For example, the NHS does not mandate its use and makes it voluntary, the use of random IDs that the NHS or the government cannot use to identify users, and not tracking users’ locations and verifying that data is held within the phone.

**Fig. 1:**
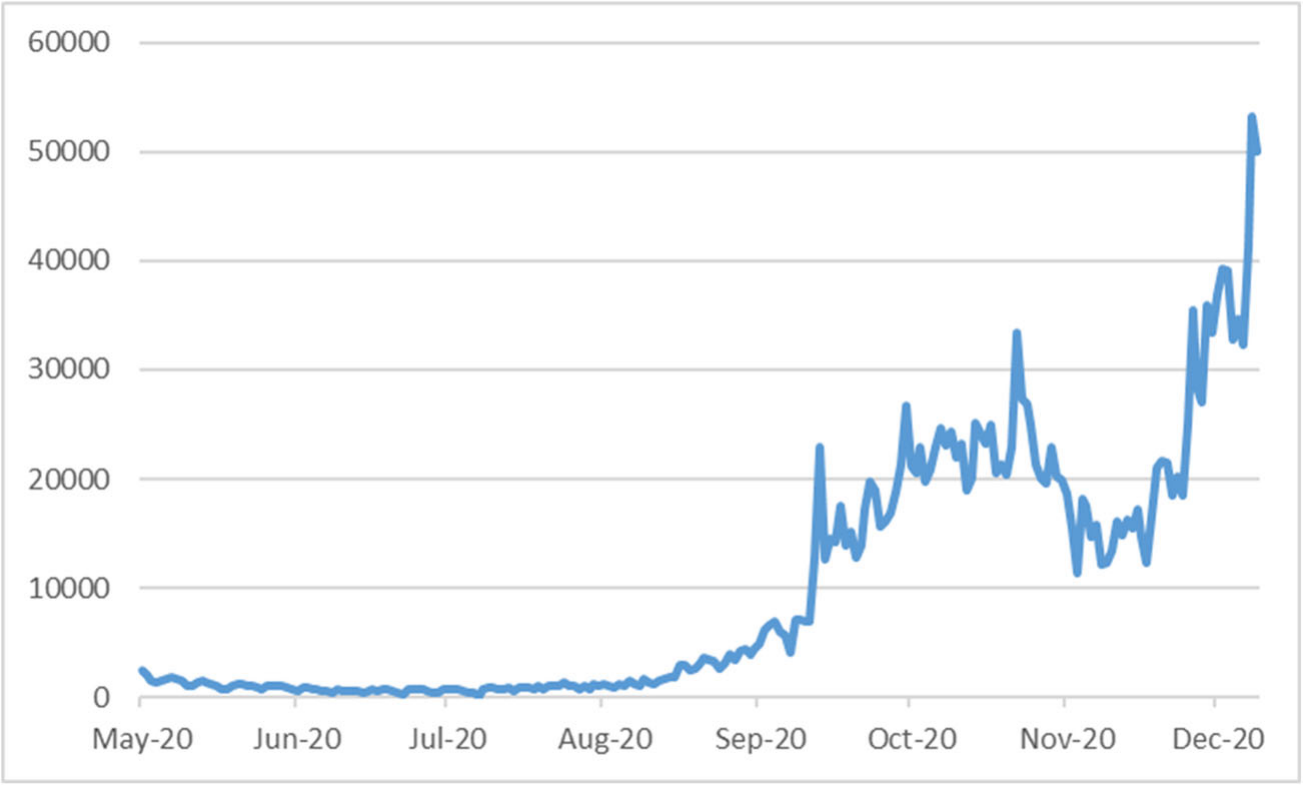
Number of New COVID-19 Cases in the UK after the introduction of NHS app(s) (May-Dec. 2020)

Nonetheless, despite all the NHS attempts to increase this app’s penetration rate by giving all necessary privacy and security assurances, the application could only attract just above 10 million users (15 % of the population) by the end of September 2020^2^. As a result, the application’s impact on controlling the virus’s spread was deemed not to be successful enough. Figure 1 presents the number of new COVID-19 cases in the UK when the application was initially introduced in May 2020 and over seven months following its introduction.

On the other hand, Qatar introduced its mobile application EHTERAZ by the Ministry of Public Health (MOPH) and Ministry of Interior (MOI) to support its fight against the pandemic earlier in April 2020. Initially, the application was running voluntarily; however, from 22nd May 2020, it became mandatory for all citizens, residents and visitors. Technically, EHTERAZ is built based on pushing information towards its users. In this regards, the application offers transmission tracking for the spread of the coronavirus, provides updated statistics related to the virus, and hotline and notification pages. When registering in the app, each user’s profile is linked to a QR code by linking it to his medical file automatically from the competent authorities.

**Fig. 2:**
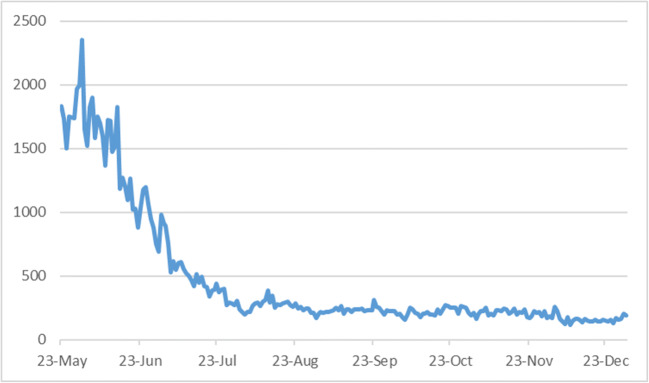
Number of new COVID-19 cases in Qatar after the introduction of the EHTERAZ app (May-Dec. 2020)

Furthermore, the application has been running on a centralised topology in which it utilises Bluetooth and GSM technologies to capture its data. Besides, given the application’s mandatory nature, more than 2.5 million users (95 % of the population) reported it. From privacy and security assurances stance, the Ministry of Public health has pointed out that the app complies with the e-Government Policies of the State of Qatar (per Council of Ministers’ Resolution No. (18) of 2010 on the Implementation of e-Government Policies) and Qatar’s Data Protection Law (Law No. 13 of 2016) concerning the privacy and protection of personal data. Besides, an informative privacy policy and commitment towards maintaining personal privacy have been provided, highlighting a few legal and governance exceptions. As a result, the application’s impact on controlling the virus’s spread appeared to be successful. Figure 2 presents how the number of new COVID-19 cases in Qatar after introducing the digital proximity tracking and tracing application has decreased between May-Dec 2020.

**Fig. 3:**
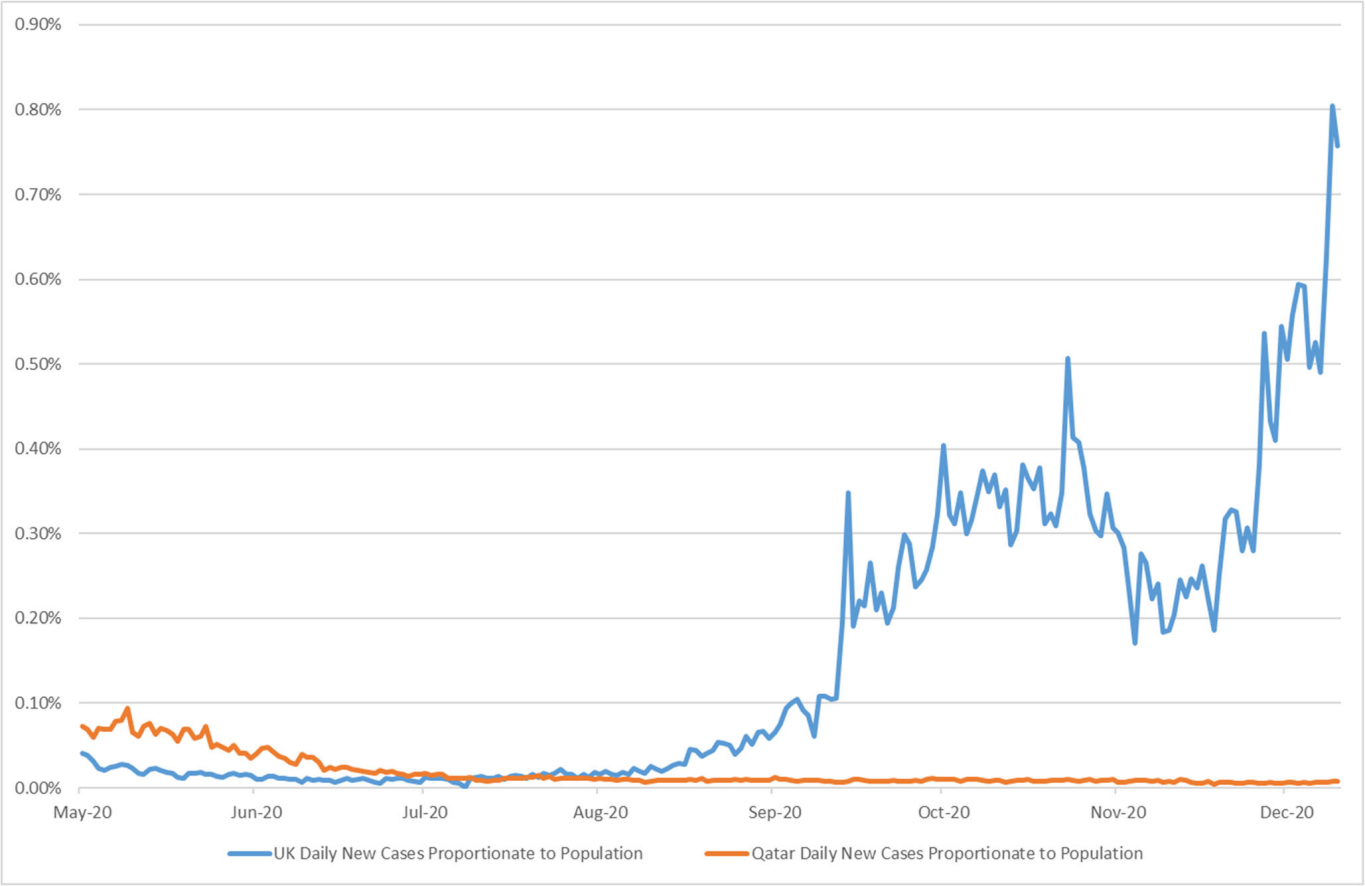
UK and Qatar daily new cases proportionate to population

When comparing both countries, it can be noticed how there have been some mixed outcomes concerning the daily number of coronavirus cases. Figure 3 depicts an indicative comparison between the UK and Qatar regarding the daily new cases proportionate to population. While in Qatar, the number of daily cases has significantly dropped and stabilised for more than four months, the UK’s daily infection cases were significantly high and on the increase. This paints a different picture regarding these AI-enabled applications’ effectiveness, explicitly concerning abiding by responsible AI principles.

### Mapping Responsible-AI Principles Against COVID-19 Digital Proximity Tracking Apps

From the responsible AI perspective, the findings corroborate how there have been considerable discrepancies for both applications resulting in different deployment impact in their fight against the coronavirus. For this study, several resources were used, including official documents, official information platforms, and associated standard repositories. While in England, the official National Health Service (NHS) and website covering the digital proximity tracking and tracing, https://covid19.nhs.uk/ has been thoroughly checked and examined, Qatar’s Ministry of Public Health website, https://covid19.moph.gov.qa, and other government websites has been utilised for the same purpose. Besides, the authors have had access to numerous publicly available resources, including official documents and reports, academically available resources, and other international independent agencies, such as World Health Organisations (WHO) and Amnesty International, to provide the necessary insights for this study. Table 2 provides a detailed comparison for mapping responsible-AI AI4people framework principles (beneficence, non-maleficence, autonomy, justice and explicability) against COVID-19 digital proximity tracking applications in UK and Qatar.

**Table 2:** Mapping AI4people framework principles against COVID-19 digital proximity tracking application in UK and Qatar

Responsible AI Principles		NHS COVID-19 Application(UK)	EHTERAZ Application(Qatar)
Beneficence	Well-Being	- A detailed justification of the benefits of using the app. - A thorough description of how the app can alert exposed users quickly. - The app allows users to report symptoms, order a coronavirus test, check in to venues by scanning a QR code, and help the NHS trace individuals with coronavirus.	- Justification of the benefits of using the app is provided. - Brief description of how the app can alert exposed users quickly. - The app helps the Ministry of Public Health trace individuals suspected or exposed, expected to be in quarantine, and tested positive for coronavirus.
	Preserving Dignity	- A detailed description of how individuals can make their decisions for using the different features within the app. This is to help in providing users with maximum freedom and minimum risk.	- A statement on how the app can help improve public health services to prevent the spread of COVID-19.
	Sustaining the Planet	- Detailed explanation on how the app can help everyone in the UK. In particular, it draws on the app ability to help the NHS understand if the virus is spreading in a particular area and so local authorities can respond quickly to stop it from spreading further to save lives. - The app can benefit those who do not have compatible smartphones and do not possess a smartphone. This can be achieved by the data captured from those who have already downloaded and used the app. This will help in understanding more about how the virus spreads. This establishes the mechanisms that will be put in place to support the country.	- An explanation of how the app can help everyone in Qatar is provided. Specifically, providing timely information on the ongoing spread levels, raising awareness and issuing recommendations to the public to protect health and safety. - To benefit from the app’s use, users will need to have compatible smartphones to download and use it. This will help understand more about how the virus spreads and establish the mechanisms that will be put in place to support the country.
Non-Maleficence	Privacy	- Strong emphasis on the importance of maintaining users’ personal privacy from the NHS and the government. - Details on how the app protects privacy and identity for all users individually and from each other are provided. - Stating third part organisations, namely, Apple and Google, have conducted a privacy review. - Users are informed that the app complies with the UK Data Protection Law and the Common Law Duty of Confidentiality. Links to these laws are provided.	- Users are informed that the app complies with the e-Government Policies of the State of Qatar (per Council of Ministers’ Resolution No. (18) of 2010 on the Implementation of e-Government Policies) and Qatar’s Data Protection Law (Law No. 13 of 2016) concerning the privacy and protection of personal data. - A privacy policy is provided. Commitment towards maintaining personal privacy when the app is used is stated. Few exceptions to this in which details have been provided.
	Security	- Strong emphasis on the importance of maintaining users’ personal security from the NHS and the government. - Details how the app guarantees security for all users individually and from each other. - Points out how specialists from the National Cyber Security Centre have been involved in the app’s design and development to ensure it is safe and secure to use. - Dedicated page for providing detailed information on the security of personal information and the data collected. - Points out explicitly the use of random IDs that the NHS or the government cannot use to identify users.	- Users are informed that their personal data, its electronic storage and transmission will be safeguarded and secured with appropriate security technologies. - Personal data will be shared with MOPH and other Government agencies in Qatar to serve the broader public’s health and safety, most efficiently and effectively, unless such sharing is prohibited by law.
	Capability Caution	- A dedicated webpage for providing detailed information on users’ security and privacy, including pointing out what the app cannot do. - The app does not access or track users’ locations. It only records distance from other phones that have the app installed. Besides, data is held within the phone.	- MOPH and the relevant Government agencies in the State of Qatar access to users location at all times during the active spread period of the COVID-19 pandemic.
Autonomy	Power to Decide	- The app does not make any decisions about any of its features. - The app is not mandatory, and users can permanently delete the app if they want. - Users can decide for themselves on whether or not to pause contact tracing within the app - Users are empowered to make the decisions to share information, especially if they had a positive COVID-19 test result to alert others.	- The application will change the health status colour from “green” to “grey” when an individual stays in close proximity to someone who tested positive for the virus. - The app is mandatory for all citizens, residents and visitors. The app will need to be running at all times when outside of their home.
Justice	Prosperity	- The use of random IDs will eliminate discrimination for all users as the NHS, and the government will not identify users. - The app is the fastest way to verify if individuals can be at risk from coronavirus. This should expedite the process of alerting and protecting others throughout the community	- Citizens and residents (above the age of 18) will need to use their Qatari identification document (QID) to register, and visitors will need to use their visit visa number. - The app notifies users when they are in proximity to a positive COVID-19 individual to protect society from its spread.
	Preserving solidarity	- The use of random IDs will eliminate any bias for datasets captured from the app used for further AI-based analysis. - The app can expedite alerting and protecting the community to minimise the coronavirus’s socio-economic impact.	- The app tracks the transmission chains of the spread of the coronavirus, providing users with accurate information and assisting the medical teams involved in providing health care when necessary
Explicability	Intelligibility	- Dedicated web pages focused on providing information on how the app work. This includes providing technical (underlying technologies) and non-technical information. - Details on the main app functions, which includes; tracing (infected individuals), alerting (coronavirus area risk), checking–in service (using QR codes for businesses), checking symptoms, supporting testing appointments and alert users when results are available, and counting down timer for self-isolation. - The app help guide is available in different explanation and support levels, including a simple/accessible read version targeting parents, guardians, and young people. Besides, it is available in twelve different languages.	- MOPH has the capabilities to trace all health status changes for all users for its app. - Details on the main app functionality. Also, the app offers to track the transmission chains of the spread of the coronavirus, providing users with accurate information and assisting the medical teams involved in providing health care when necessary. Furthermore, the app provides updated statistics related to the virus as well as hotline and notification pages. - When registering in apps, each user’s profile is linked to a QR code by linking it to his/her medical file automatically from the competent authorities. The app is available in Arabic and English languages.
	Accountability	- Technical auditing capabilities are available for all features in the app. - The app is administered and owned by the Department of Health and Social Care. - All entities involved in the development of the app are named. The government leads technical authority on cybersecurity, and the National Cyber Security Centre has provided support in an advisory role.	- MOPH can trace and perform necessary audits for all health status changes for all users of its app. - The app is owned and operated by the Ministry of Public Health of Qatar (“MOPH”) and the relevant government entities.

### Diagnostic Sentiment Analysis for AI-Enabled COVID-19 Digital Proximity Tracking and Tracing Engagement Strategies

As part of the NLP lexicon-based approach, the emergence of sentiment analysis has offered the opportunity to examine opinions, thoughts closely, and behaviours based on textual data (Sun et al., 2017; Ji et al., 2015; Liu, 2012). Positive, negative and neutral sentiments scoring have provided a valuable perspective regarding data polarity (Sun et al., 2017; Ravi & Ravi, 2015). Twitter, the microblogging platform, has offered endless opportunities to examine much-needed insights, specifically policymakers, to help build better understanding and engagement (Kang et al., 2017), especially during the pandemic, in which direct access to primary empirical data can be minimal. For this study, Twitter data has been captured to closely examine how the NHS and MOPH, and other associated entities, have aligned the ethical perspective in their official communications and engagement with the public about these digital proximity applications. Thus, two sets of tweets were scrapped from Twitter, covering those administered by official/formal agencies in both countries during the period between 1st March until 31st December 2020. These official tweets are expected to provide valuable insights regarding how both countries have attempted to promote their digital proximity track and trace apps to capture the ethical perspective and the sentiment through their engagement with the public. For the EHTERAZ app, tweets were captured in Arabic and English, official country communications, languages to provide appropriate coverage. A total of 4205 tweets were collected in both languages. Keywords used in the search include “EHTERAZ” for English tweets and “تطبيق_احتراز” for Arabic tweets. Geolocation was carefully considered in this process, focusing only on tweets originating from Qatar. After extracting the official ones, 85 official tweets were captured and then analysed accordingly.

Similarly, for the NHS proximity tracking and tracing app, official tweets were captured over two periods to cover the NHS proximity tracking and tracing app (i.e. NHSx and NHS COVID19 apps). For the first period, 9394 tweets between 1st March until 23rd September 2020. These tweets covered the times when the NHS introduced its first centralised proximity tracking and tracing app supported by NHSx. Keywords utilised in the search included “NHSx”, “NHS tracking and tracing”, and “NHS digital”. On the other hand, the NHS has launched on 24th September 2020 its new decentralised dedicated proximity tracking and tracing NHSCOVID19 app. 15,192 tweets were captured between 24th September to 30th December 2020, with the name of the app, “NHS COVIID19app”, itself was used as the main keyword search. All tweets covering the two periods were collected in the English language. Besides, the geolocation was taken into considerations focusing only on tweets originating from England. The extraction process covered several iterations to ensure that only official tweets are appropriately utilised during both periods, and this resulted in 467 tweets that were examined and analysed consequently. Table 3 provide a summary of the steps and iterations included in this process.

**Table 3:** Twitter iterations and filtering process

	UK (England)		Qatar
Name of official app	NHS alpha and beta versions	NHS COVID19 APP	EHTERAZ
Tweets Scrapping Period	1st March − 23rd September 2020	24th September − 30th December 2020	1st March – 30th December 2020
Language	English	English	English and Arabic
Keywords Used for search	NHSx, NHS tracking and tracing, NHS digital	NHSCOVID19app	EHTERAZ, تطبيق_احتراز
Geolocation	England	England	Qatar
Source of Tweets	NHS, England Primary Care Trusts (PCTs)	NHSCOVID19 app, England Primary Care Trusts	All official Qatar Ministries
Total number of tweets	9394	15,192	4205
Total number of official tweets	47	490	88
Total number of direct official tweets (with no reply)	25	435	88

#### EHTERAZ App sentiment outcomes

**Fig. 4:**
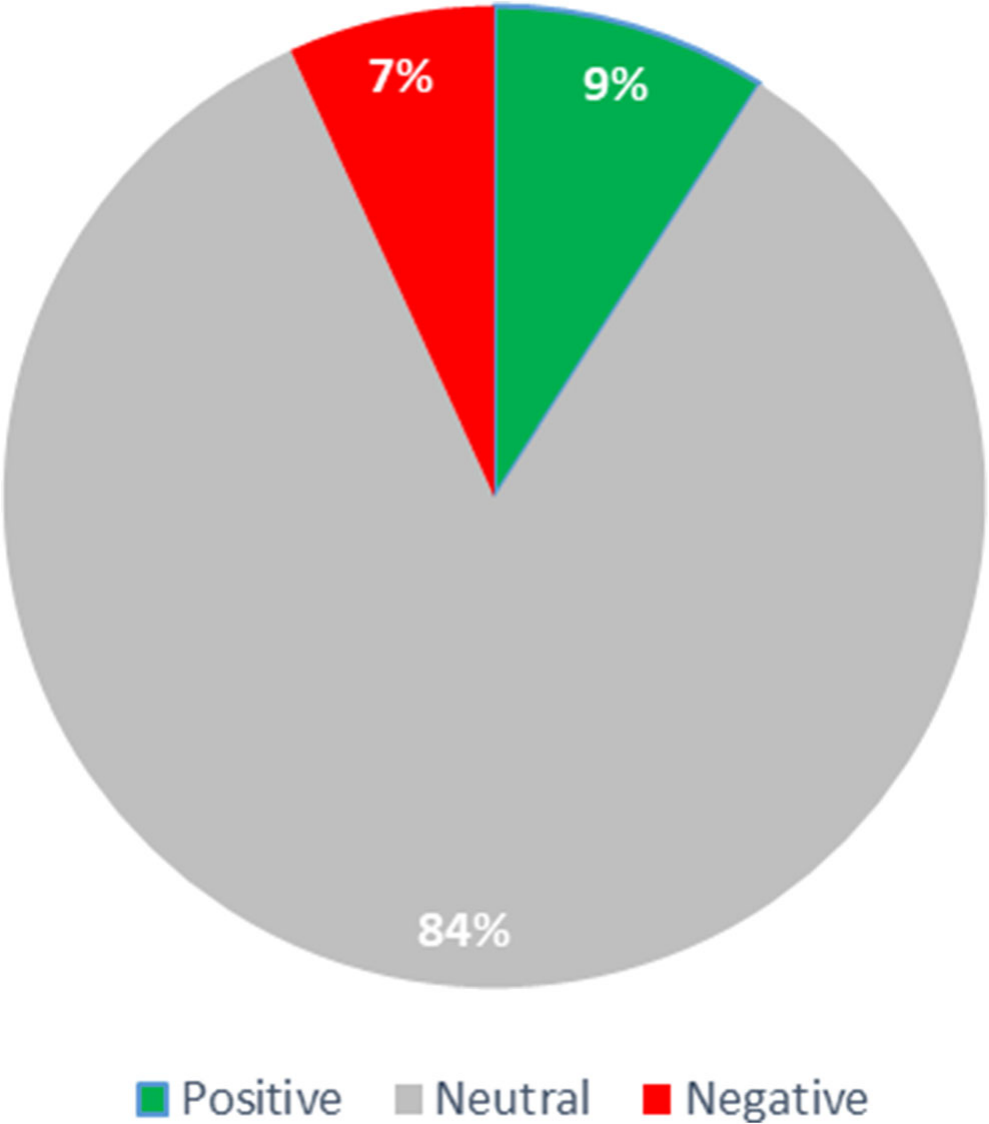
EHTERAZ tweets sentiment analysis

For the EHTERAZ tweets sentiment analysis assessment, a careful examination of the tweets in Arabic and English was conducted. It was noted that the official tweets, in both languages, were the same. Therefore, to avoid any unnecessary duplications, English language tweets were considered for this analysis. Positive, negative and neutral sentiments scoring have been adopted to offer the needed perspective regarding data polarity for official Qatari ministries engagement on this social media platform. Out of the total 85 official tweets captured over six months, 74 tweets came as neutral, 6 tweets negative sentiment, and 8 tweets were positive sentiments. Figure 4 depicts the distribution of these tweets, in which the engagement sentiment about the EHTERAZ app appears to be neutral. Such a result appears to be associated and aligned with the mandatory nature of using this digital proximity application.

**Fig. 5:**
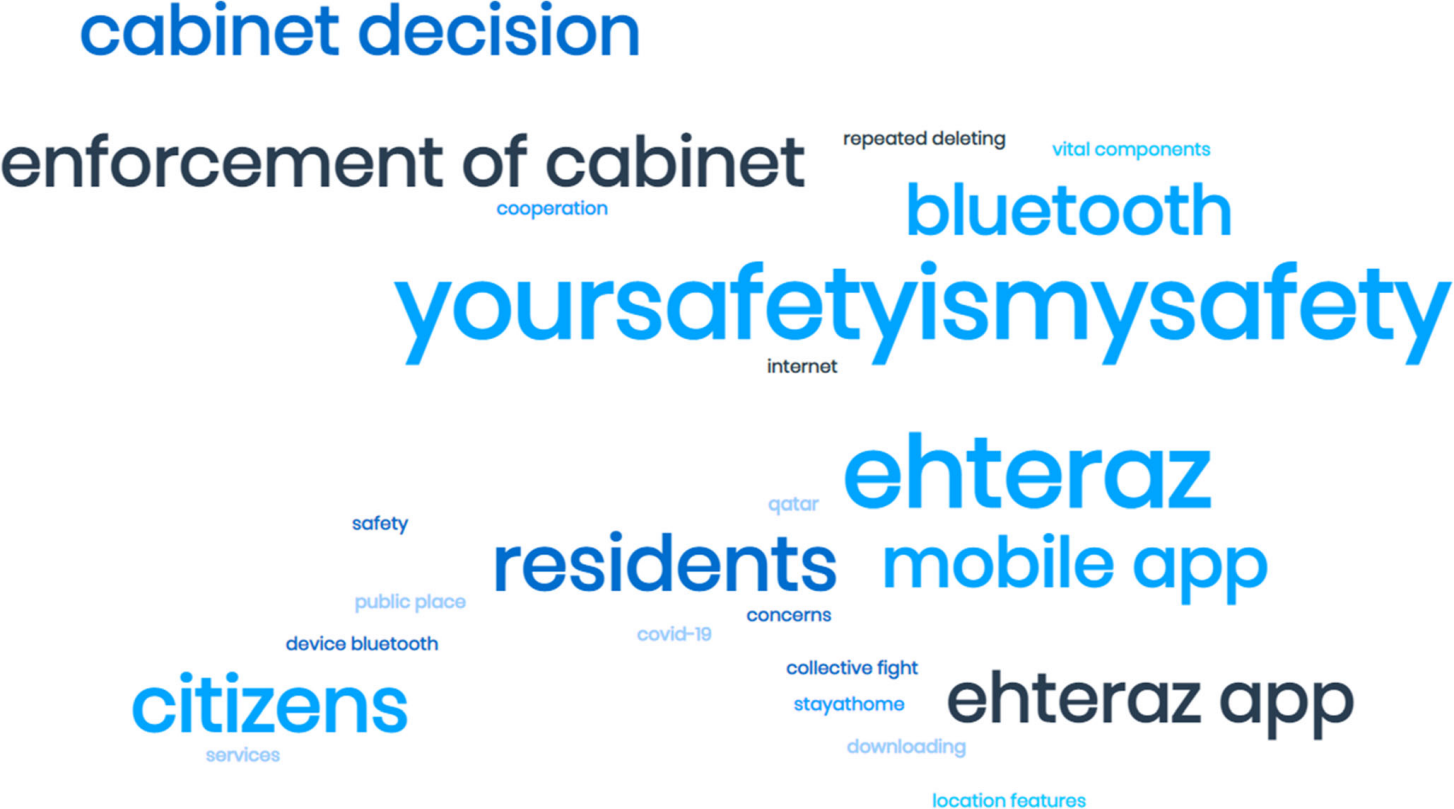
Word cloud for EHTERAZ tweets

When analysing the word frequencies for the EHTERAZ related tweets, the word cloud generated, in Fig. 5, details how many of these tweets were directed towards informing citizens and residents about the importance of installing and activating the app. While the hashtag “your safety is my safety” was the most common phrase among the terms, others referred to how the government, via cabinet decision, signified the need to continue using this application to help everyone stay safe.

From a responsible AI perspective, it is noteworthy to point out how the mandatory installation and activation of the EHTERAZ app, manifested by the government, has offered little to align the ethical outlook in their direct engagement on this social media platform. In this regards, much emphasis has been directed towards how the app can help improve individual and community safety and tracking the spread of COVID-19. Additionally, the app’s users’ data is confidential and accessible by relevant, specialised teams when necessary.

#### NHS App Sentiment Outcomes

**Fig. 6:**
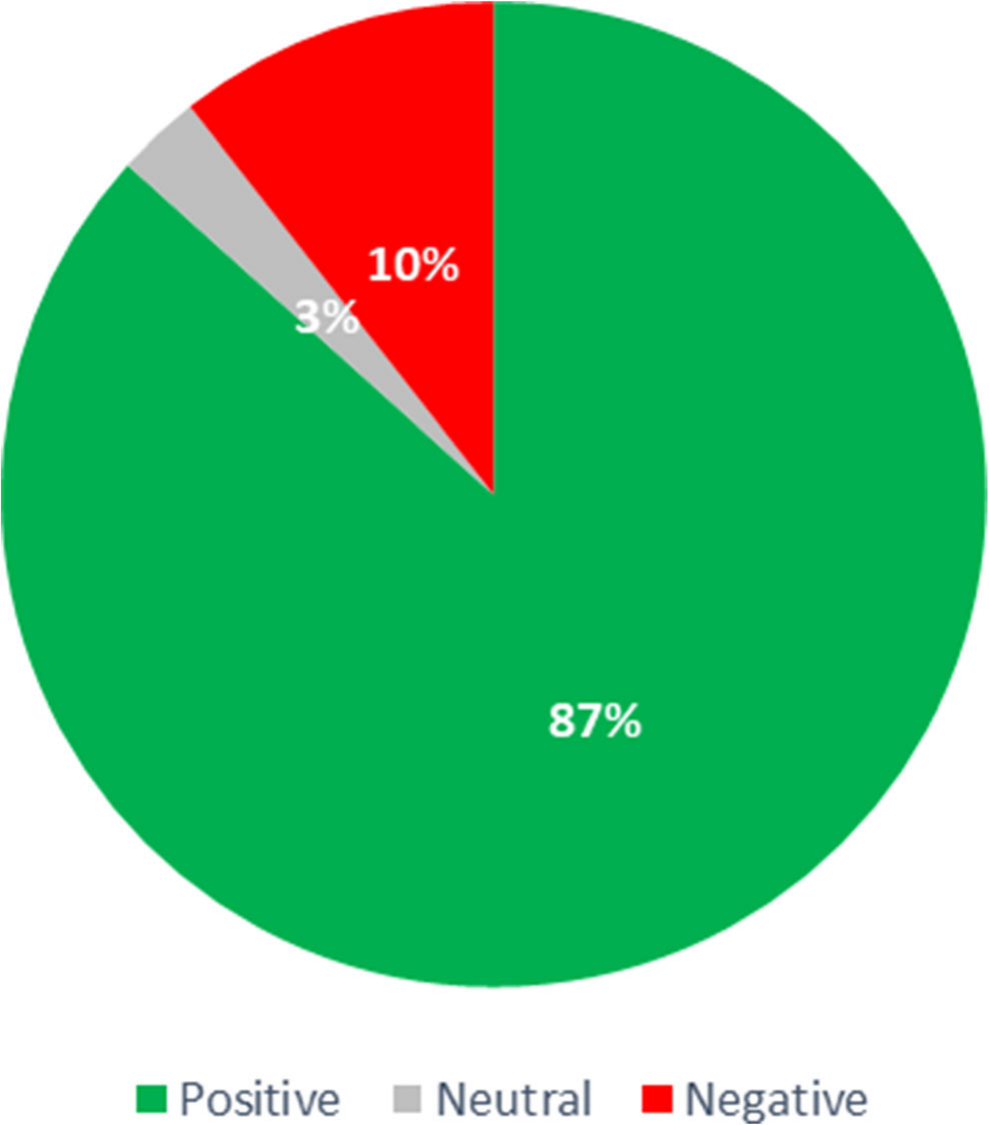
NHS COVID-19 tweets sentiment analysis

A similar process has been followed for conducting the tweets sentiment analysis assessment for the NHS COVID19 tweets. All English language tweets were considered for this analysis while considering the geolocation and the origins of these tweets. Positive, negative and neutral sentiments scoring have been adopted to offer the needed perspective regarding data polarity for official NHS engagement on this social media platform. Out of the total 435 official tweets captured over three months, 377 tweets came as positive, 12 tweets neutral sentiments, and 46 tweets were negative sentiments. Figure 6 provides the distribution of these tweets, in which the engagement sentiment about the NHS COVID19 app appears to be positive. The outcome of this analysis verifies how the NHS aimed to maintain a positive engagement strategy to encourage and facilitate the use of this digital proximity application.

**Fig. 7:**
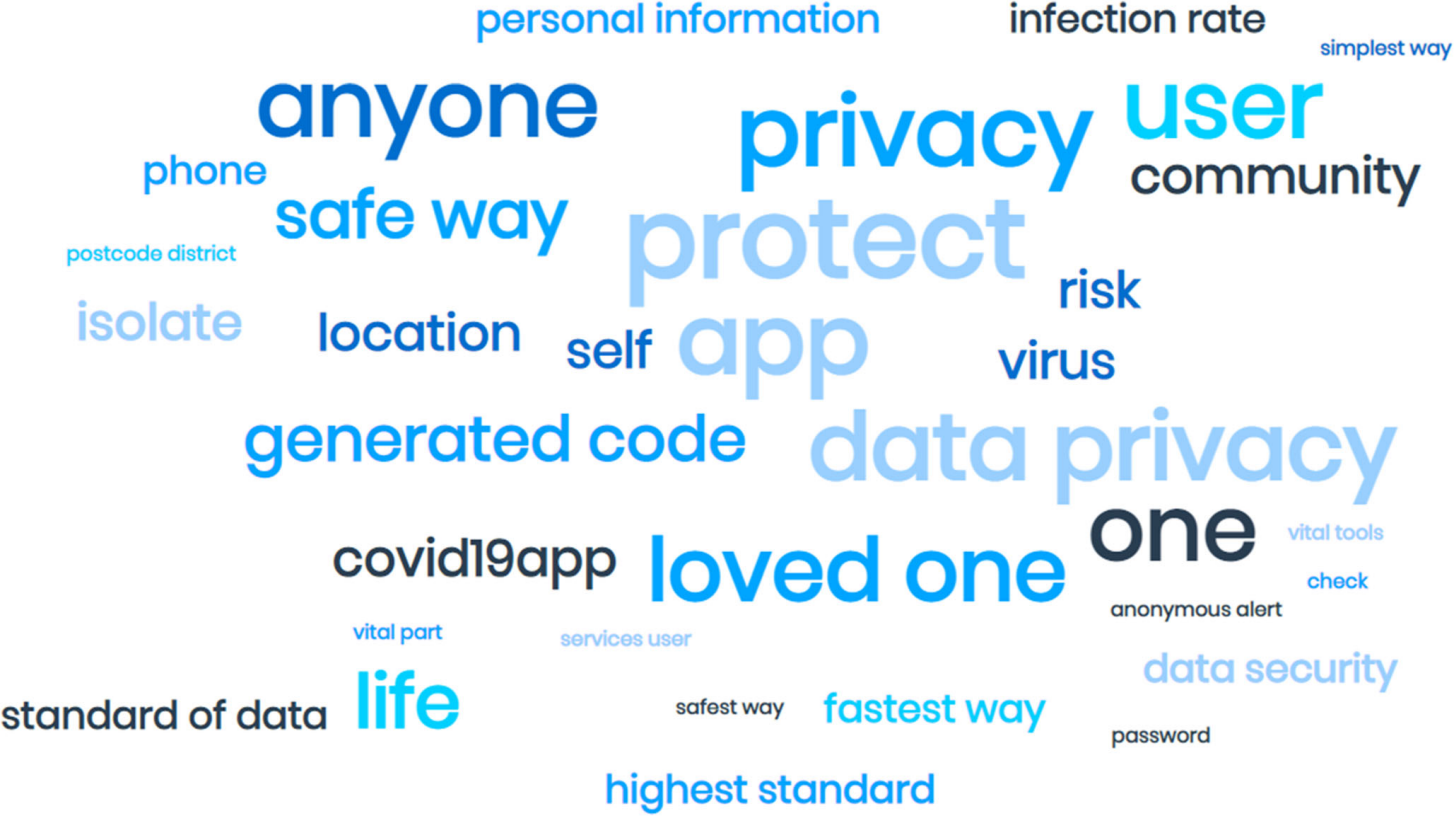
Word cloud for NHS COVID-19 tweets

For the word frequencies analysis for the NHS COVID19 app tweets, the word cloud generated, in Fig. 7, detailed how many of these tweets were directed towards positively engaging with users about this app’s contribution towards safeguarding public health, safety and security. The words “privacy” and “protect” were the most common words highlighting the importance of the need to be active and help protect the community, stay alert, and stop the infection rates from rising.

Furthermore, from a responsible AI perspective, it was evident how the NHS opted to maintain an ethical perspective throughout the engagement activities on this microblogging platform. In this regard, the thematic analysis for the NHS COVID-19 tweets reveals how the NHS and affiliated primary care trusts adopted high compliance with these technologies’ ethical requirements. From the AI4people framework perspective, the findings reveal a close alignment against all five principles. Table 4 summarises findings for the captured tweets against all five AI4People responsible AI principles.

**Table 4:** Thematic analysis for NHS COVID-19 tweets mapped against responsible AI principles framework

AI4People responsible AI principles				
Beneficence	Non-maleficence	Autonomy	Justice	Explicability
- Protect yourself - Protect loved ones - Save lives - Keep safe - Learn about virus - Fighting virus	- Users are anonymous - Cannot track location - Secure - Data privacy - Data security - Private by design	- Data held on the phone - Available to download - Cannot force to self-isolate	- Tracking virus - Alert anyone at risk - Cannot force to identify - Protect community - Stop infections from rising	- Requires first half of postcode - Low Bluetooth energy - Venue check-in - Available in 12 languages - Managed by NHS

## Discussion

It is evident how Artificial Intelligence technologies can offer a promising approach towards tackling societal challenges, specifically in healthcare. In the coronavirus pandemic context, countries have taken different measures and approaches to protect their citizens’ health. This includes utilising innovative technologies, such as AI-enabled digital proximity tracking and tracing applications, to support these measures. Such potential is hoped to help control the pandemic’s spread to withstand the social and economic challenges in many countries (Von Wyl et al., 2020). While the two countries in this study have deployed such applications, their successes, including adoption rate, penetration and impact, have varied. One potential explanation can be related to ethical, security and privacy considerations. As such, this study examined the considerations of responsible AI in the deployment of COVID-19 digital proximity tracking and tracing applications in the State of Qatar and the United Kingdom. The research findings highlighted some level of divergence for the two apps in terms of responsible AI compliance, considerations and the impact of controlling the virus’s spread. As such, the findings verified that the UK NHS COVID-19 app has a relatively high compliance rate compared to Qatar’s EHTERAZ. Consequently, one would expect that the UK app would have a higher penetration rate and impact than the Qatari counterpart. Counterintuitively, the NHS COVID-19 app has a relatively low penetration level, resulting in limited contributions towards controlling the virus’s spread. On the other hand, the EHTERAZ app achieved a very high level of users’ penetration resulting in a higher level of success in significantly reducing the virus’s spread. It is clear that the success of the apps in curbing the spread of the virus is a function of its adoption rate and that the adoption rate, in the context of the COVID-19 pandemic, can be achieved more by stricter mandate (EHTERAZ) than by stricter compliance with responsible AI considerations. Gostin et al. (2020) identified the dilemma between voluntary and enforcement compliance. On the one side, there is public health interest, which requires protection from the virus. However, on the other side, imposing restrictions, tracking, and tracing individuals can raise questions about compromising society’s ethical and privacy values. In the UK, it seems the government’s ability to mandate adoption is much more stringently restricted by regulatory frameworks such as the General Data Protection Regulation, which went into effect in 2018. Qatar’s mandatory approach, although cognizant of the country’s Protection of Personal Data Law of 2016, appears to be perhaps founded on the Islamic principle of “preventing harm precedes procuring gain”, thus mandating the use of the app to curb the pandemic (i.e., preventing the harm) takes precedence over procuring the gain of full compliance with the ethical and responsible requirements. Judging on the outcomes of the two approaches in stopping the spread of the virus, it seems the approach taken by Qatar achieved a better success. However, one cannot generalise that the UK’s approach to be somewhat inherently flawed, because of the low penetration rate, despite high compliance with the ethical AI framework. This could also be attributed to a lack of complete awareness of such features by the citizens, active counter push from anti-anything government propaganda, or perhaps fatigue from the social distancing and other measures.

Nonetheless, these diagnostic findings demonstrate an unresolved challenge debating the needed balance between protecting public health in its fight against the pandemic while complying with the societal and ethical requirements. Floridi et al. (2018) claimed how the public demands a clear realisation of the benefits of AI technologies while appropriately maintaining a robust risk mitigation approach in order to adopt them. Furthermore, Gasser et al. (2020) argued how decision-makers have the responsibility to provide efficient and effective legal and ethical measures for utilising digital health systems. This validates the need to set up the mechanisms towards building societal and a health perspective resilience to appreciate responsible AI’s role in these difficult times. To do so, cultural and geographical differences become vital to preserving fairness, equality, and inclusion. Therefore, a cross-disciplinary collaboration involving AI experts, medical professionals, policymakers, NGOs and other stakeholders will be needed in order to raise awareness and to promote the benefits of these technologies while at the same time demonstrating its depth and the width for the ethical considerations and responsibility.

## Conclusions

For digital health technologies, AI algorithms’ ability to learn from existing healthcare data offers unparalleled opportunities for many healthcare providers; ranging from automating numerous healthcare services to augmenting and supporting medical professionals’ diagnosis and decisions (Chin-Yee & Upshur, 2019; Harerimana et al., 2018; He et al., 2019). Nonetheless, exploitation of these algorithms has raised many concerns in many areas, including the ethical side. While it appears that AI has enormous potential to revolutionise healthcare services, a considerable amount of efforts concerning ethical and legal work are yet to be addressed (Schönberger 2019). Challen et al. (2019) and He et al. (2019) claimed that while establishing rules and regulatory measures can offer some governance and compliance mechanisms, observing end-user ethical rights and appreciating the societal value remain a challenge.

The aim of this study was focused on examining responsible AI’s considerations in the deployment of COVID-19 digital proximity tracking and tracing applications in two countries, the State of Qatar and the United Kingdom. AI4people framework principles were employed to offer the required ethical lens for mapping these AI-based applications. Furthermore, secondary data resources were utilised to conduct the required examination in this study. Official social media engagement sentiment analysis and official documents, platforms, and associated standard repositories were exploited for this purpose to offer the required diagnostic insights, understanding and necessary triangulation. The obtained findings highlighted how the two countries examined in this study had different outcomes and impact. This underlines the need for obtaining a practical and contextual view contributing to the discourse on responsible AI in healthcare. Therefore, striking a balance between responsible AI requirements and managing the pressures towards fighting pandemics will be required.

From implications for the research viewpoint, this study utilised the AI4people framework and sentiment analysis of official engagement activities on Twitter to examine responsible AI consideration in the context of COVID-19 digital proximity tracking and tracing applications. In particular, the study makes a valuable and unique contribution in advancing the knowledge in the applicability and maturity of AI applications from an ethical perspective. By investigating these features, this research has offered valuable insights on realising the challenge in balancing between fulfilling responsible AI requirements while managing the pressures in fighting against pandemics. In addition, this study offers a broad understanding of the consideration of responsible AI in the context of healthcare. In particular, the taxonomy presented in section two contributes to providing the needed acumens of various ethical challenges facing the exploitation of this innovative tool while at the same time addressing relevant mitigating strategies that could help in overcoming such challenges. Furthermore, the study findings offer valuable insights into the need to implement successful and positive engagement strategies to change users’ perceptions of the ethical perspective associated with the adoption and diffusion of innovative technologies, such as AI.

For practice, this study highlighted that AI-based applications’ deployment requires careful considerations for their practical and contextual views. Therefore, all involved stakeholders will need to take a proactive role in implementing responsible AI applications. In the context of digital proximity tracking and tracing, public healthcare providers and policymakers will be required to work closely with third-party entities to design, develop, and deploy these applications effectively. By doing so, this will help in developing the required maturity and at the same time moving forward towards developing and harmonising an integrated responsible AI consideration across all processes. As demonstrated in this study, utilising secondary data can help provide the practical insights needed when access to direct primary empirical data can be limited. Simultaneously, social media data exploitation in examining sentiments and polarity can offer valuable and valid insights to unveil how engagement and communication strategies require careful consideration.

Nonetheless, as with any work, there are several inherent limitations in this study. The examination for AI-based digital proximity tracking and tracing applications has been based on existing secondary data resources, including reports and information available through the applications and Twitter data. As such, this can offer limited insights about the consideration of responsible AI within. It is accepted that the ability to operationalise the measures in this study may not be ideal. While this can be justified given the limited availability of empirical data, it can ultimately impact this study’s overall generalisability. Nonetheless, the work remains focused on presenting a diagnostic perspective. Besides, the adopted AI4People framework offers focused insights towards these applications. Therefore, this requires further investigation through direct and active engagement with relevant stakeholders to capture more in-depth and practical insights. Furthermore, the study has only examined two existing digital proximity tracking and tracing applications, limiting the findings’ generalisability. Therefore, future research should aim to target other countries’ applications to perform a cross-comparison and perform the required measurements. The unit of analysis of this study was not aimed at examining cross-cultural, social and political issues but rather on responsible AI’s perspective. While this is an early attempt to consider responsible AI in a pandemic context, this paper has nevertheless addressed a relevant research gap on acknowledging the role of responsible AI in mitigating ethical risks for the deployment of COVID-19 digital proximity tracking and tracing applications.

The insights obtained from this study offer stakeholders an opportunity to realise the need for building communications bridges internally and externally. It is hoped that this can advance the debate to move forward towards developing and harmonising an aspirational mindset that can help to utilise AI technologies successfully. These conversations can ultimately help acknowledge responsible AI’s role in mitigating its ethics and promote fairness, transparency, prosperity, accountability, and explainability. Furthermore, this study highlights that the debate over responsible AI is still unresolved and will most likely resurface with future health-related challenges. Hopefully, with less intensity as more maturity might be achieved in terms of embedding ethical design in future AI applications as a result of advancement in ethical AI scholarship and practice.

## Footnotes

1
https://covid19.nhs.uk/.

2 https://www.computerweekly.com/news/252489711/NHS-Covid-19-app-exceeds-10-million-downloads-but-has-teething-troubles.
